# A dominant-negative *IFNGR1* variant reveals broad immune cell sequestering of IFN-**γ**

**DOI:** 10.1172/JCI186799

**Published:** 2025-04-15

**Authors:** Samantha Chan, Mai B. Margetts, Longfei Wang, Jack Godsell, Josh Chatelier, Belinda Liu, Charlotte A. Slade, Andrew Brett, Kasha P. Singh, Vanessa L. Bryant, Lauren J. Howson

**Affiliations:** 1 Immunology Division, Walter and Eliza Hall Institute of Medical Research, Parkville, Australia.; 2 Department of Medical Biology, The University of Melbourne, Parkville, Victoria, Australia.; 3 Department of Clinical Immunology and Allergy, Royal Melbourne Hospital, Parkville, Victoria, Australia.; 4 Department of Medicine, The University of Melbourne, Parkville, Victoria, Australia.; 5 Department of Infectious Diseases and Immunology, Austin Health, Heidelberg, Victoria, Australia.; 6 Genetics and Gene Regulation Division, Walter and Eliza Hall Institute of Medical Research, Parkville, Victoria, Australia.; 7 Department of Respiratory Medicine and Sleep Disorders, Royal Melbourne Hospital, Parkville, Victoria, Australia.; 8 Department of Gastroenterology, Royal Melbourne Hospital, Parkville, Victoria, Australia.; 9 Department of Infectious Diseases, The Peter Doherty Institute for Infection and Immunity, Melbourne, Victoria, Australia.; 10 Victorian Infectious Diseases Service, Royal Melbourne Hospital, Parkville, Victoria, Australia.

**Keywords:** Genetics, Immunology, Cellular immune response, Genetic diseases

**To the Editor:** IFN-γR1 deficiency is a form of Mendelian susceptibility to mycobacterial disease (MSMD) caused by partial or complete loss-of-function variants in *IFNGR1* (1). Complete IFN-γR1 deficiency is autosomal recessive (AR) and characterized by complete penetrance, early onset, and severe infections (1, 2). Partial IFN-γR1 deficiency can be AR or autosomal dominant (AD) and typically has later onset, with less severe infections (1, 2). Dominant-negative IFN-γR1 deficiency is caused by variants in *IFNGR1* exon 6 that result in a truncated receptor lacking both the intracellular internalization motif and the STAT1 docking site. This leads to surface accumulation of nonsignaling IFN-γ receptors that compete with WT receptors (1, 3). However, the implications of these nonsignaling receptors on the bioavailability of IFN-γ have not yet been explored. Here, we report an AD *IFNGR1* variant in a family with MSMD and demonstrate the ubiquitous nature of IFN-γR1 expression and the capacity for dominant-negative IFN-γR1 variants to sequester IFN-γ on the cell surface.

Whole-exome sequencing confirmed a heterozygous *IFNGR1* variant c.817delA (p.I273fs) present in family members with clinical disease (Figure 1A, see Supplemental Figure 1 and Supplemental Table 1 for patients’ clinical details; supplemental material available online with this article; https://doi.org/10.1172/JCI186799DS1). This variant occurs within exon 6 of *IFNGR1*, where previous dominant-negative variants have been reported (3).

IFN-γR1 is moderately expressed by almost every cell type in healthy individuals, and, in our patients, all PBMC subsets overexpressed IFN-γR1 (Figure 1B), having a 5–9-fold higher expression compared with individuals who were healthy (Supplemental Figure 2B). This is consistent with previously reported exon 6–truncated *IFNGR1* variants (3) showing overexpression on monocytes and T cells, but furthers our understanding of the ubiquitous nature of patients’ IFN-γR1 overexpression to also encompass NK cells, B cells, and γδ T cells, and particularly high expression on MAIT cells.

It is suggested that IFN-γ signaling may be rescued by the addition of high-dose IFN-γ in dominant-negative IFN-γR1 deficiency (4). We observed a small dose-response effect to a maximum of one-fold increase in IFN-γR1^WT/I273fs^ monocyte pSTAT1 at 10 ng/mL IFN-γ that did not increase to healthy levels (7-fold) with increasing dose (Figure 1C). Addition of 0.1 ng/mL IFN-γ induced a maximum 1-fold increase in IFN-γR1^WT/I273fs^ monocyte LPS-induced TNF production, which did not increase to healthy levels (4-fold) with increasing dose (Figure 1D). RNA-seq analysis confirmed that IFN-γR1^WT/I273fs^ monocyte sensitivity to IFN-γ could not be rescued with high-dose exposure across downstream gene targets of IFN-γ signaling (Figure 1E).

We next investigated IFN-γR1 binding kinetics by culturing PBMCs with IFN-γ and measuring IFN-γR1 and IFN-γ by flow cytometry. Upon binding IFN-γ, the WT receptor decreased 2.5-fold at the surface and 1.4-fold intracellularly (Supplemental Figure 2C). The IFN-γR1^WT/I273fs^ cells, with an overall higher level of IFN-γR1 baseline expression, showed impaired surface (0.7-fold decrease) and intracellular (0.5-fold decrease) decreases, suggesting an impaired degradation due to absence of the internalization domain in these variant receptors. As expected, IFN-γ was undetectable on IFN-γR1^WT/WT^ monocytes, due to the WT receptor internalization following IFN-γ binding (Figure 1F). However, IFN-γR1^WT/I273fs^ monocytes showed a dose-dependent increase in surface IFN-γ at concentrations up to 1,000 ng/mL (Figure 1F). IFN-γ was highest on monocytes, but all PBMC subsets exhibited detectable dose-dependent increases (Figure 1G). We then measured IFN-γ cytokine-receptor dissociation by preincubating patient PBMCs with IFN-γ and measuring surface IFN-γ on monocytes over time. We observed prolonged detection of IFN-γ on the cell surface, with a dissociation half-life of 2 hours (Figure 1H).

Broad sequestering of IFN-γ on the patients’ cell surface has the potential to reduce systemic IFN-γ bioavailability in affected patients. This may include endogenous IFN-γ, providing an explanation for the low plasma IFN-γ in patients with AD IFN-γR1 deficiency, while IFN-γ plasma levels in AR IFN-γR1 are typically either moderate (partial deficiency) or high (complete deficiency) (5, 6). It may also impact the bioavailability of exogenous IFN-γ, such as the recombinant therapy used to treat acute refractory mycobacterial infection. IFN-γ treatment has been reported to be effective in certain cases of dominant-negative IFN-γR1 anecdotally (summarized in Supplemental Table 2); however, no studies have directly assessed the efficacy of IFN-γ therapy for patients with dominant-negative IFN-γR1 deficiency.

In summary, we have demonstrated impaired IFN-γ signaling in AD IFN-γR1 deficiency that cannot be rescued by high-dose IFN-γ in vitro. This is potentially due to prolonged surface retention of IFN-γ by ubiquitously overexpressed truncated IFN-γR1, which establishes an inaccessible reservoir of IFN-γ sequestered on cell surfaces in dominant-negative IFN-γR1 deficiency.

## Supplementary Material

Supplemental data

Supporting data values

## Figures and Tables

**Figure 1:**
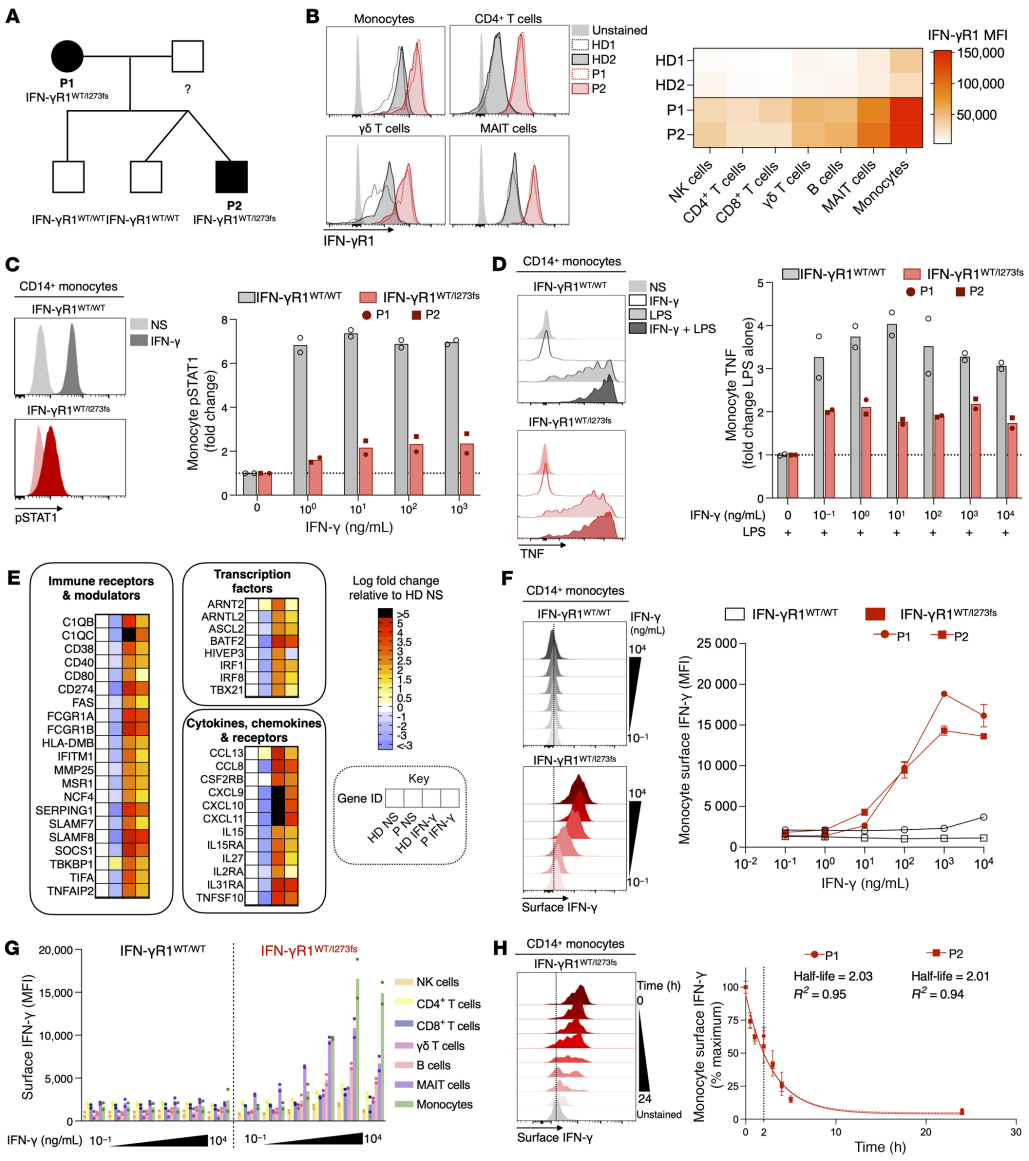
IFN-γR1 is ubiquitously overexpressed and sequesters IFN-γ on the surface of patients’ cells. (**A**) Familial segregation of the *IFNGR1* c.817delA (p.I273fs) variant. (**B**) Expression of IFN-γR1 on PBMC subsets. (**C**) pSTAT1 staining of PBMCs stimulated with IFN-γ and gated on monocytes. (**D**) PBMCs were cultured with LPS and a 10-fold dilution series of IFN-γ. (**E**) RNA-seq of differentially expressed immune genes. Surface IFN-γ detected on (**F**) monocytes and (**G**) PBMC subsets following incubation with IFN-γ. (**H**) IFN-γ dissociation from monocytes over time. The line represents nonlinear regression and the dashed line the dissociation half-life. Error bars represent SD between technical duplicates. HD, healthy donor; MAIT, mucosal-associated invariant T (cell). NS, no stimulation; P, patient.

## Version 1 04/15/2025

Electronic publication
