# Supplementary material for: A dominant-negative IFNGR1 variant reveals broad immune cell sequestering of IFN-γ
Source: J Clin Invest. 2025 Apr 15;135(8):e186799. doi: 10.1172/JCI186799 (PMC11996848; doi:10.1172/JCI186799)
Supplement: Supplemental data [file jci-135-186799-s113.pdf]

Supplementary Materials for:

A dominant negative *IFNGR1* variant reveals broad immune cell sequestering of IFN-γ

Samantha Chan, Mai B. Margetts, Longfei Wang, Jack Godsell, Josh Chatelier, Belinda Liu, Charlotte A. Slade, Andrew Brett, Kasha P. Singh, Vanessa L. Bryant & Lauren J. Howson

Correspondence: Samantha Chan (samantha.chan@mh.org.au) & Lauren J. Howson (howson.l@wehi.edu.au)

This file contains:

Case Summary
Methods
Acknowledgements
Authorship Contributions Statement
Supplementary Figures 1 and 2
Supplementary Tables 1 and 2
Supplementary References

Supplementary Materials

Case Summary

The proband (P1) had a local infection caused by BCG vaccination at age 12, which gradually resolved without treatment (Supplementary Figure 1). In 1987, at age 25, she presented with weight loss, night sweats and erythema nodosum and was subsequently diagnosed with severe disseminated mycobacterial infection in the bone, lymph nodes, and lung. The patient was hospitalized for several months while undergoing *M. avium* complex (MAC) treatment with azithromycin, rifabutin, and ethambutol. She remained on MAC treatment for an extended course of 15 years, after which all antimicrobials were ceased. She remained well until age 56, when she re-presented with anterior uveitis and a rash over her scalp and lower limbs. MAC was isolated from a biopsy of the skin lesion. The patient was treated with rifabutin, ethambutol, and clarithromycin (later switching to azithromycin after developing nausea). She responded well to a two-year treatment course and has since remained on a prophylactic course of azithromycin (See Supplementary Table 1 for full clinical history). The patient has not received adjunct IFN-γ therapy to treat any mycobacterial infection.

The proband's son (P2) was diagnosed in 2004, at age 4, after developing pneumonia with pneumatoceles (Supplementary Figure 1). BCG vaccination had not been part of his immunization schedule. A nuclear scan revealed avid lesions in his ribs, mandible, clavicle, skull, and radius. A rib biopsy was taken, and MAC was isolated. The patient underwent MAC treatment consisting of 2 years of azithromycin, rifabutin, and ethambutol, after which no prophylaxis was required. He has not had any further mycobacterial infections to date and has not received adjunct IFN-γ therapy to treat any mycobacterial infection. See Supplementary Table 1 for full clinical history.

Methods

Sex as a biological variable

Our study involved both a male and female patient with age- and sex-matched healthy donors. Similar findings are reported for both sexes.

Study design

Patients were identified by clinicians at the Royal Melbourne Hospital Department of Immunology & Allergy, and their clinical histories, including details of their Mendelian susceptibility to mycobacterial disease (MSMD) diagnosis, were established from their medical records. Age- and sex-matched healthy donors were recruited through the volunteer blood donor registry at the Walter & Eliza Hall Institute of Medical Research (WEHI). Healthy donor age, sex, and medical history was self-reported. See Supplementary Figure 1A for overview of patients' MSMD clinical course.

Human sample processing

Blood samples were collected via venipuncture. PBMC were isolated by density gradient centrifugation using Ficoll-Paque Plus (Cytiva). Cells were cryopreserved in liquid nitrogen.

Whole exome sequencing

DNA was extracted from cryopreserved PBMCs using DNeasy kit blood & Tissue (Qiagen) following the manufacturer's instructions. Samples were submitted to the Australian Genome Research Facility (AGRF) for 20 gb Twist Clinical Alliance Exome sequencing. Whole exome sequencing was performed using the Illumina NovaSeq X Plus platform. Raw sequencing data were processed using the Genome Analysis Toolkit (GATK) Best Practices pipeline (1), which includes the following steps:

1. Mapping raw reads to the human reference genome (GRCh38/hg38)
2. Marking duplicates
3. Recalibrating base quality scores
4. Variant calling using HaplotypeCaller (2)
5. Joint calling
6. Filtering variants with Variant Quality Score Recalibration (VQSR)

Variants were annotated using ANNOVAR (3) and Ensembl Variant Effect Predictor (VEP) (4). An R shiny app was developed for interactive variant filtering (R version 4.0.5). Variants were prioritized based on predicted variant consequence (using VEP, SIFT (5), PolyPhen-2 (6) and CADD (7) scores) and the allele frequency, with highest priority given to rare or novel variants.

See Supplementary Figure 1B for summary of sequencing results and Supplementary Figure 1, C and D for variant gene and protein consequence.

Antibodies and staining reagents

All antibodies used were commercially available and validated for specificity by the manufacturer. BD Horizon BUV395 anti-human CD19 (SJ25C1), BD Horizon BV786 anti-human IFN-γ (4S.B3), BD OptiBuild BUV615 anti-human CD14 (63D3.rMAb), and BD OptiBuild BUV805 anti-human CD161 (HP-3G10) were purchased from BD Biosciences. Allophycocyanin (APC) anti-human CD3 (SK7), APC/Cyanine7 anti-human CD16 (B73.1), Brilliant Violet 605 anti-human TCR Vα7.2 (3C10), Brilliant Violet 650 anti-human CD4 (OKT4), Pacific Blue anti-human CD3 (SK7), PE anti-human CD119 (IFN-γR1 α chain) (GIR-94), PE/Dazzle 594 anti-human TNF-α (MAb11), PE/Cyanine7 anti-human CD4 (SK3), PerCP/Cyanine5.5 anti-human CD8 (SK1), and Ultra-LEAF Purified anti-human IFN-γ (B27) were purchased from Biolegend. FITC anti-human TCRγ/δ (REA591) was purchased from Miltenyi Biotech. PE anti-human phospho-STAT1 (Tyr701) (KIKSI0803) was purchased from ThermoFisher Scientific (eBioscience). Dead cells were excluded using the viability dye Zombie Aqua Fixable Viability Kit (BioLegend) according to the manufacturer's instructions.

Flow cytometry

For flow cytometry cell staining, cells were thawed and stained with viability dye for 10 min at room temperature, followed by antibodies diluted in PBS with 10% heat-inactivated FBS (Sigma) for 20 min on ice. Flow cytometry data was collected on a Cytek Aurora spectral flow cytometer. Data were analyzed using FlowJo software (BD, v10). See Supplementary Figure 2A for flow cytometry gating strategy.

IFN-γ activation of pSTAT1 assay

PBMCs were stained with viability dye before being pulsed with human recombinant IFN-γ (STEMCELL Technologies). Sample was cultured (at 37°C and 5% CO_2 in humidified incubator) in cell culture media (RPMI-1640 medium supplemented with 10% heat-inactivated FBS and antibiotics) for 30 min. Cells were then immediately fixed with eBioscience IC Fixation buffer for 30 min followed by permeabilization with ice-cold methanol for 30 min at 4°C. Intracellular antibodies were then incubated for 45 min at room temperature.

LPS activation assay

PBMCs were cultured in cell culture media and pulsed with human recombinant IFN-γ (STEMCELL Technologies) (at indicated concentrations) and 1 μg/mL LPS-EK (InvivoGen). Samples were cultured for 1 h prior to addition of Brefeldin A (BFA) (eBioscience) (when intracellular staining) and cultured for 18 h.

Following stimulation, cells were collected and stained with viability dye for 10 min followed by surface antibodies for 20 min on ice. For intracellular staining, cells were then fixed and permeabilized using the eBioscience Intracellular Fixation & Permeabilization Buffer Set (ThermoFisher Scientific). Intracellular antibodies diluted in permeabilization buffer were then incubated for 45 min at room temperature.

IFN-γ dissociation assay

PBMCs were pulsed with 1 μg/mL IFN-γ and cultured in cell culture media for 1 hour. Cells were washed and cultured in cell culture media for a period of 24 h. Aliquots of cells were collected at indicated timepoints, stained with live/dead fixable dye, and fixed in eBioscience IC Fixation buffer. Fixed cells were then surface stained and analyzed by flow cytometry.

RNA-seq

Monocytes were isolated using EasySep Human Monocyte Isolation Kit (STEMCELL Technologies) according to the manufacturer's instructions. The monocytes were then incubated with IFN-γ for 6 h. RNA was extracted using an RNAmicro kit (Qiagen) according to the manufacturer's instructions. Samples were submitted to AGRF for RNA quality check on an Agilent Bioanalyzer 2100, library preparation using Illumina Stranded mRNA Prep. Sequencing was performed using an Illumina NovaSeq X Plus with 150 bp paired end read.

RNA-seq analysis

Raw sequencing files were trimmed and low-quality reads filtered using Trim Galore and aligned to the Ensembl Human GRCh38 genome using STAR aligner (v2.3.5a). Consensus transcripts were assembled using StringTie and number of reads mapped using FeatureCounts. The resulting gene count matrix was uploaded to the Degust web tool (8) using the voom/limma method for testing significant differential expression analysis. A false discovery rate (FDR) of 0.05 cut-off

was implemented calling differentially expressed genes. Heatmaps show log2 fold change in gene expression relative to healthy (no stimulation), averaged between biological replicates (n = 2).

Analysis

Analysis and graphing were performed using Prism (GraphPad Software, v10).

Study approval

Ethical approval for this study was granted by the Human Research Ethics Committees of Melbourne Health (project ID: 2009.162) and WEHI (project ID: 10/02). Written, informed consent was obtained from all participants, in accordance with the Declaration of Helsinki prior to their participation in the study.

Data availability

The whole-exome sequencing data have been deposited in the NCBI Sequence Read Archive (SRA) under the BioProject Accession Number: PRJNA1183433. RNA-seq raw sequencing files have been deposited in the NCBI SRA under the BioProject Accession Number: PRJNA1205773. For values for all figures see the Supporting Data Values file.

Acknowledgements

We sincerely thank all the study participants. We would like to acknowledge Sylvia Tsang, Maureen Forde, and the Volunteer Blood Donor Registry (WEHI) for assistance with sample collection. We acknowledge the use of the services and facilities of AGRF and WEHI flow cytometry facility. This research was supported by: NHMRC Investigator Grant 2007884 (L.J.H.), WEHI Scientific Excellence PhD Scholarship (S.C.), and Sir Clive McPherson Family (V.L.B.).

Authorship Contributions Statement

Conceptualization: S.C. and L.J.H.; Investigation: S.C., M.B.M. and L.J.H.; Formal analysis: L.W. and L.J.H.; Resources: S.C., J.G., J.C., B.L., A.B., K.P.S. and V.L.B.; Data curation: S.C. and L.J.H; Project administration: S.C. and L.J.H.; Funding acquisition: L.J.H.; Writing – original draft: L.J.H.; Writing – review & editing: S.C., C.A.S., V.L.B. and L.J.H.

Supplementary Figures

Supplementary Figure 1. Patients clinical course of mycobacterial infection, exome sequencing summary and variant details.

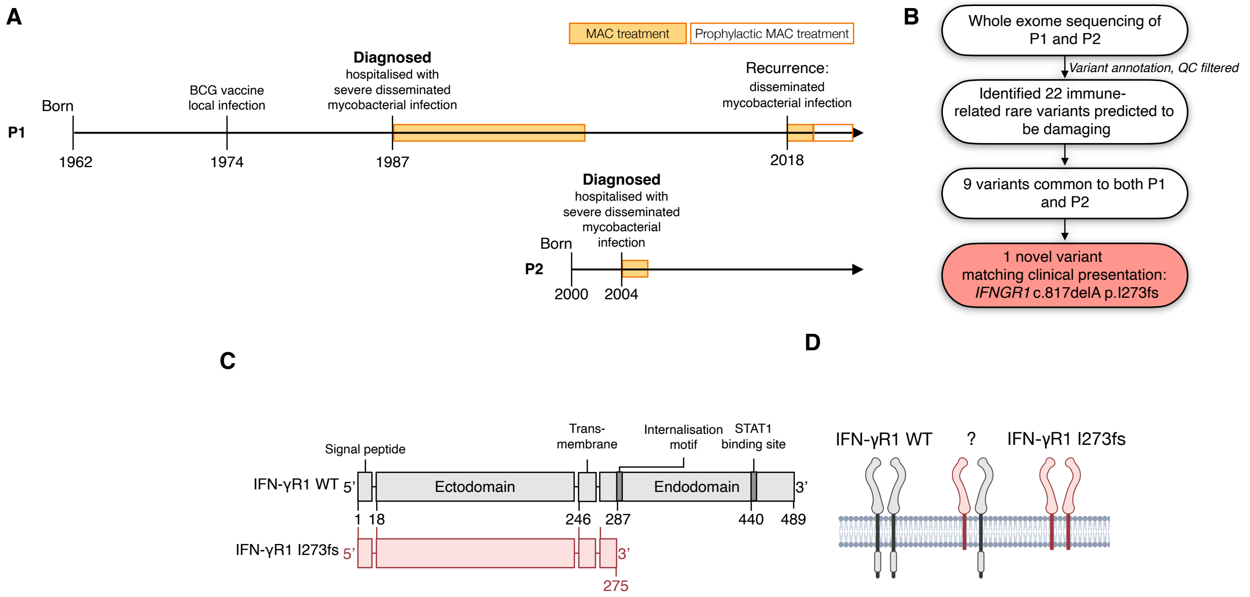

(**A**) Clinical timeline of mycobacterial infections and treatment for P1 and P2. (**B**) Schematic of whole exome sequencing variant detection pipeline. (**C**) Graphical representation of the IFN-γR1 protein and domains for WT and truncated I273fs. Numbers indicate amino acid number. (**D**) Illustration of WT (grey) and I273fs (red) IFN-γR1 dimers on cell surface. BCG, Bacillus Calmette-Guerin; MAC, *Mycobacterium avium* complex; QC, quality control.

Supplementary Figure 2. Flow cytometry gating strategy and IFN-γR1 expression.

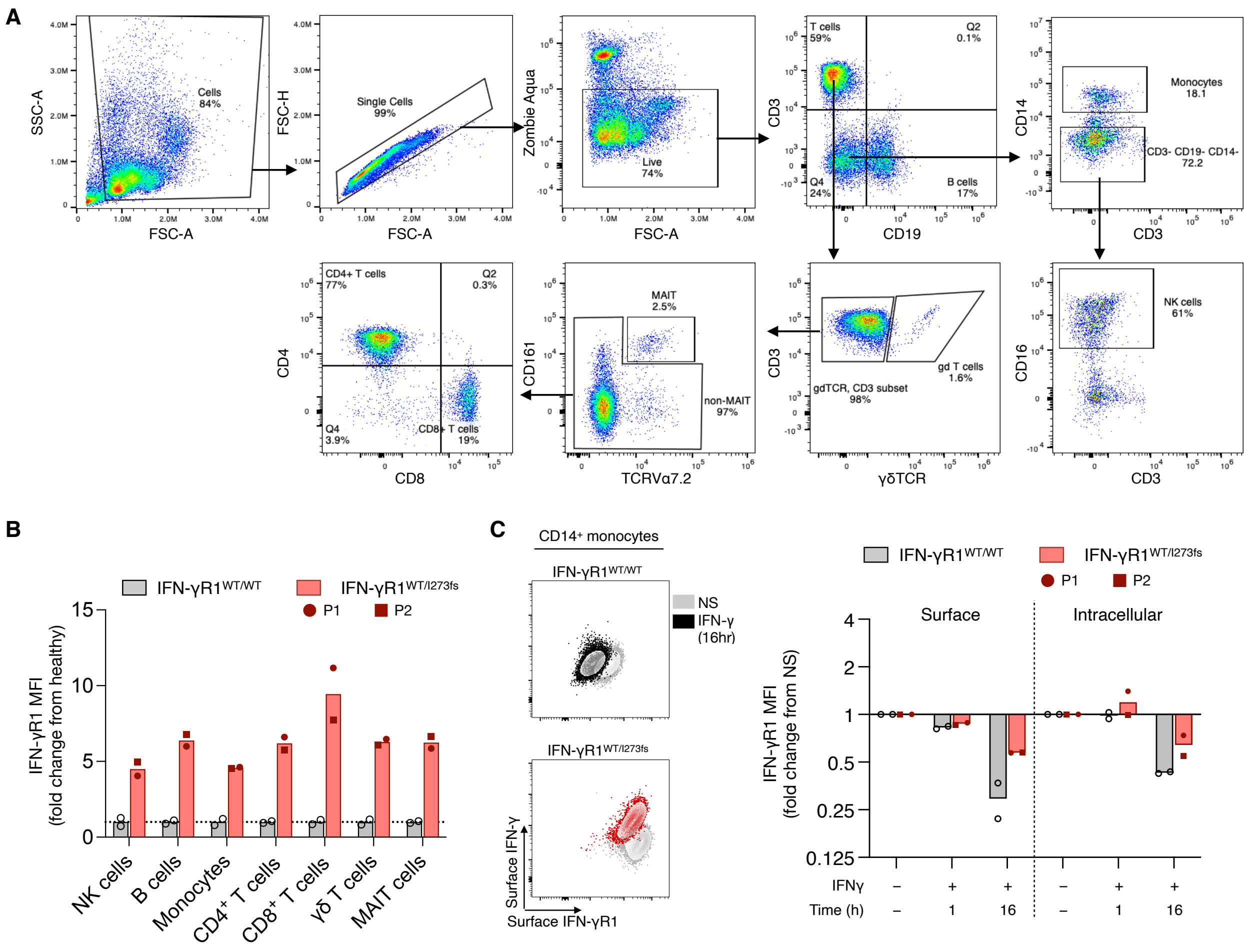

(**A**) Plots show the gating strategy of PBMC samples based on forward and side scatter, single cells, live, $CD19^+$ B cells, $CD14^+$ monocytes, $CD16^+$ NK cells and $CD3^+$ T cells (then gating on: $CD4^+$ T cells, $CD8^+$ T cells, MAIT cells, and γδ T cells). (**B**) IFN-γR1 fold change expression on PBMC subsets, relative to healthy donors. (**C**) IFN-γR1 fold change expression (surface and intracellular) for monocytes in response to high-dose IFN-γ (10 μg/mL) incubated for 1 or 16 h, relative to NS sample. FSC-A, forward scatter (area); MAIT, mucosal associated invariant T (cell); NS, no stimulation; TCR, T cell receptor; SSC-A, side scatter (area).

Supplementary Tables

Supplementary Table 1. Summary of patient clinical history.

	**Proband**	**P2**
**Age** (years), **sex**	62, Female	23, Male
**Age at diagnosis** (years)	25	4
**Initial presentation**	B symptoms, hepatosplenomegaly, bony lesions, subcutaneous nodules	Pneumonia with pneumatoceles, bony lesions
**Index mycobacterial infection**	Disseminated *M. avium* complex	Disseminated *M. avium* complex
**Anti-microbial tx for index infection** (duration)	Azithromycin, rifabutin, ethambutol (15 years)	Azithromycin, rifabutin, ethambutol (2 years)
**Recrudescent infection:** age, presentation, tx (duration)	Age: 56<br>Scalp and thigh lesions; mycobacterial DNA isolated from biopsy<br>Tx: Azithromycin, rifabutin, ethambutol (2 years)	None to date
**Anti-microbial prophylaxis**	Azithromycin (following recrudescent infection)	Nil
**Co-morbidities**	Bilateral anterior uveitis<br>Glaucoma<br>Osteoarthritis (bilateral total knee replacements)<br>Squamous cell carcinoma<br>Gastric oesophageal reflux disease	Bronchiectasis<br>Chronic rhinosinusitis with nasal polyps<br>Asthma<br>Granuloma annulare
**Other medications**	Topical ocular triamcinolone and brinzolamide<br>Meloxicam<br>Rabeprazole	Budesonide/formoterol<br>Mepolizumab

Tx, treatment.

Supplementary Table 2. Summary of reported cases of adjunct IFN-γ therapy for AD IFN-γR1 deficiency.

Year	No. of patients	Summary	Reference
1999	6	Total of 18 patients with AD IFN-γR1 deficiency, Of which 6 received IFN-γ therapy. Of these 6 cases, resolution of infection was attributed to the addition of recombinant IFN-γ in 3 individuals. In the other 3 cases, the clinical response to IFN-γ is not detailed, or its specific impact cannot be ascertained because IFN-γ therapy was initiated immediately with antimicrobial treatment upon diagnosis of infection (patient descriptors, further data not provided).	(9)
2000	Not provided	Reported that in patients with intact IFN-γ receptors, including the AD IFN-γR1, and disseminated MAC or other non-tuberculosis mycobacteria infections, that subcutaneous IFN-γ has produced very favorable results, with a cure rate of 60% (data unpublished).	(10)
2014	1	Attributed the addition of IFN-γ therapy to antimicrobial therapy regime as resolving BCG infection in a 1-year-old child with AD IFN-γR1	(11)

AD, autosomal dominant; BCG, Bacillus Calmette-Guérin; MAC, *Mycobacterium avium* Complex.

Supplementary References

1. Van der Auwera GA, Carneiro MO, Hartl C, Poplin R, Del Angel G, Levy-Moonshine A, et al. From FastQ data to high confidence variant calls: the Genome Analysis Toolkit best practices pipeline. *Curr Protoc Bioinformatics.* 2013;43(1110):11 0 1- 0 33.
2. Poplin R, Ruano-Rubio V, DePristo MA, Fennell TJ, Carneiro MO, Van der Auwera GA, et al. Scaling accurate genetic variant discovery to tens of thousands of samples. *bioRxiv.* 2018:201178.
3. Wang K, Li M, and Hakonarson H. ANNOVAR: functional annotation of genetic variants from high-throughput sequencing data. *Nucleic Acids Res.* 2010;38(16):e164.
4. McLaren W, Gil L, Hunt SE, Riat HS, Ritchie GR, Thormann A, et al. The Ensembl Variant Effect Predictor. *Genome Biol.* 2016;17(1):122.
5. Ng PC, and Henikoff S. SIFT: Predicting amino acid changes that affect protein function. *Nucleic Acids Res.* 2003;31(13):3812-4.
6. Adzhubei IA, Schmidt S, Peshkin L, Ramensky VE, Gerasimova A, Bork P, et al. A method and server for predicting damaging missense mutations. *Nat Methods.* 2010;7(4):248-9.
7. Rentzsch P, Witten D, Cooper GM, Shendure J, and Kircher M. CADD: predicting the deleteriousness of variants throughout the human genome. *Nucleic Acids Res.* 2019;47(D1):D886-D94.
8. Powell D. Zenodo; 2015:An interactive web-tool for RNA-seq analysis.
9. Jouanguy E, Lamhamedi-Cherradi S, Lammas D, Dorman SE, Fondaneche MC, Dupuis S, et al. A human IFNGR1 small deletion hotspot associated with dominant susceptibility to mycobacterial infection. *Nat Genet.* 1999;21(4):370-8.
10. Holland SM. Treatment of infections in the patient with Mendelian susceptibility to mycobacterial infection. *Microbes Infect.* 2000;2(13):1579-90.
11. Takeda K, Kawai T, Nakazawa Y, Komuro H, Shoji K, Morita K, et al. Augmentation of antitubercular therapy with IFNgamma in a patient with dominant partial IFNgamma receptor 1 deficiency. *Clin Immunol.* 2014;151(1):25-8.
